# Editorial: Macrophage Metabolism and Immune Responses

**DOI:** 10.3389/fimmu.2020.01078

**Published:** 2020-05-29

**Authors:** Héctor Rodríguez, Rafael Prados-Rosales, José Luis Lavín, Massimiliano Mazzone, Juan Anguita

**Affiliations:** ^1^Inflammation and Macrophage Plasticity Lab, CIC bioGUNE-BRTA (Basque Research and Technology Alliance), Derio, Spain; ^2^Department of Preventive Medicine and Public Health and Microbiology, Autonoma University of Madrid, Madrid, Spain; ^3^Bioinformatics Unit, CIC bioGUNE-BRTA (Basque Research and Technology Alliance), Derio, Spain; ^4^Laboratory of Tumor Inflammation and Angiogenesis, Center for Cancer Biology, VIB, Leuven, Belgium; ^5^Laboratory of Tumor Inflammation and Angiogenesis, Department of Oncology, Center for Cancer Biology, KU Leuven, Leuven, Belgium; ^6^Ikerbasque, Basque Foundation for Science, Bilbao, Spain

**Keywords:** macrophage, metabolism, infection, disease, immune response

Macrophages are key components of the immune response to infectious organisms, with a dual role eliminating infectious agents by phagocytosis while mediating defensive and inflammatory pathways. On the other hand, macrophages are also involved in the late response to infections by heavily contributing to tissue debris clearance and “wound” repair. The importance of the metabolic status of these immune cells on their responses has been overlooked for decades. However, recent advances in metagenomics and high-throughput metabolic profiling have partially unveiled the role of metabolism in macrophage responses. New evidence suggests that the metabolic status and specific metabolic pathways are instrumental for the modulation of macrophage responses to the ever-changing environment that these cells face *in vivo*. For example, a role of metabolism in reactive oxygen species production in response to pathogens has been long known. Furthermore, macrophage transition from an anti to a pro-inflammatory phenotype seems to be at least partially mediated metabolic reprogramming from fatty acid oxidation to glycolysis. Recent studies have also shown metabolic plasticity during long-term responses to vaccine formulations of individual pathogen-associated molecular patterns. The capacity of macrophages to be trained, an emerging concept describing the “learning” capacity of innate immune cells, seems to be also underpinned by metabolic switches. This Research Topic aims to shed light on our current understanding of the role of metabolism in macrophage biology and versatility focusing on the immune response to infectious agents.

Two of the articles in the topic are specifically dedicated to the role of macrophage metabolism during infection. In a review, Ramond et al., discuss the ability of some bacterial pathogens to manipulate macrophage metabolic programs to survive intracellularly and how mitochondria could play a paramount role by regulating cellular metabolism and immune responses against these pathogens. Although studies addressing this interplay need further refinement to reflect *in vivo* conditions, they encourage the implementation of host-directed therapies for clinical infections where manipulation of metabolic responses through this organelle can help to resolve infections. More specifically, to clarify the observed susceptibility to infection of bone marrow macrophages (BMMs) from CBA mice by two species of Leishmania, Bezerra de Menezes et al., performed a differential proteomic analysis of infected macrophages and found that host responses related to oxidative stress and iron metabolism might dictate the intracellular fate of this pathogen.

Different articles in the topic are focused on the importance of metabolic adjustments for macrophage activation. In a review by Diaz-Bulnes et al., the authors address the phenomenon of metabolic rewiring in macrophage activation, and how those metabolic modifications allow those cells to adapt to changes in the environment (such as hypoxia or oxidative stress) maintaining their pro-inflammatory and anti-microbial activities. This response is coordinated by different metabolic mechanisms. In this review, they illustrate the relation of the metabolic and phenotypic plasticity of macrophages with hypoxia and endoplasmic reticulum stress, and how they can relate to human pathology and chronic inflammation. Two different reviews in the topic summarized, using different point of views, the relevance of macrophage metabolic activation with the focus on metabolic disease. Daemen and Schilling, highlight the multiple and relevant connections between obesity and macrophage metabolic activation. The article initially covers macrophage dynamics and diversity in liver and adipose tissue, both crucial areas for the study of metabolic diseases. The review also summarizes the available knowledge regarding the role of macrophage metabolic regulation for metabolic diseases and, in particular, obesity, also addressing the importance of niche determinants for this metabolic tuning and for the overall function of macrophages. Furthermore, Daemen and Schilling describe future challenges in the topic and suggest new therapeutic possibilities based on macrophage targeting in order to treat metabolic disorders. Meanwhile, the role of lipids in the metabolic reprogramming of macrophages is reviewed by Batista-Gonzalez et al.. They summarize the available knowledge regarding the role of lipids in macrophage plasticity from the importance of the differential fatty acid metabolism in the dichotomic, *in vitro* endorsed, M1 (pro-inflammatory) to M2 (anti-inflammatory) transition, down to the role of lipids on macrophage activation. In this review, the authors summarize new research challenging the old consensus that defines M1 as aerobic glycolytic cells and fatty acid oxidation as characteristic of M2 macrophages suggesting a more dynamic metabolic reprogramming. Finally, they suggest possible therapeutic interventions targeting lipid metabolism in order to prevent the activation of macrophages. Also in relation to lipids, original research by Sohrabi et al., highlight the relationship of trained innate immune responses to the reprogramming of different cellular metabolic pathways like those related to the synthesis of cholesterol and fatty acids. It is specifically explored the role of Liver Receptor X (LXR), a key regulator of these pathways, whose activation promotes a proinflammatory trained immunity phenotype in human monocytes.

In an original research article focused on the interface of nutrition and macrophage activation, Erkelens et al. experimentally confirmed previous observations of the effect of Vitamin A in the modulation of inflammation within the gut. In particular, they showed that the vitamin favors the anti-inflammatory state of intestinal tissue resident macrophages within the gut. Furthermore, the article shows that Vitamin A increases Dectin-A expression levels in macrophages under homeostatic conditions. The authors further show that Dectin-A induction provokes the release of pro-inflammatory cytokines by macrophages. Based on these results, Erkelens et al. suggest that Dectin-A-induced signaling might provide a switch from an anti-inflammatory to a pro-inflammatory state of the macrophage when needed. Finally, another original research article is presented by Yang and Ding, demonstrating that MEK1/2 inhibitors unravel the interferon response via interferon regulatory factor 1 (Irf1) signaling in macrophages. They pinpoint a deficiency in *Irf1* that abolishes the interferon response and prevents macrophages from reprogramming into an immunostimulatory phenotype. A MEK1/2 inhibitor enables IRF1-mediated interferon signature response in macrophages, while a combination of inhibitor and agonist of *Irf1* have potential as therapy.

The manuscripts included in this Special Issue highlight and expand our understanding of the adaptative capacity of innate immune cells in response to inflammatory signals and pinpoint the critical importance that metabolic rearrangements play in the response kinetics of these first responders of the immune system. They also highlight the variability and intricacies of these metabolic adaptations, shedding light into novel targets of intervention in order to fine tune the response of macrophages to different pathological conditions.

## Author Contributions

HR, RP-R, JL, MM, and JA edited the topic. HR, RP-R, and JL wrote the manuscript. JA and MM reviewed and corrected the manuscript.

## Conflict of Interest

The authors declare that the research was conducted in the absence of any commercial or financial relationships that could be construed as a potential conflict of interest.

